# The bioinformatics analysis of RIOX2 gene in lung adenocarcinoma and squamous cell carcinoma

**DOI:** 10.1371/journal.pone.0259447

**Published:** 2021-12-02

**Authors:** Bingqing Sun, Hongwen Zhao

**Affiliations:** Department of Pulmonary and Critical Care Medicine, First Affiliated Hospital of China Medical University, Shenyang, Liaoning, China; Virginia Commonwealth University, UNITED STATES

## Abstract

Lung cancer is characterized by high morbidity and mortality rates, and it has become an important public health issue worldwide. The occurrence and development of tumors is a multi-gene and multi-stage complex process. As an oncogene, ribosomal oxygenase 2 (RIOX2) has been associated with a variety of cancers. In this article, we analyzed the correlation between RIOX2 expression and methylation in lung cancer based on the databases including the cancer genome atlas (TCGA) (https://portal.gdc.cancer.gov/) and the gene expression omnibus (GEO) (https://www.ncbi.nlm.nih.gov/geo/). It was found that RIOX2 is highly expressed in lung adenocarcinoma (LUAD) and lung squamous cell carcinoma (LUSC) tissues, whose expression is negatively correlated with its methylation level. In this regard, methylation at cg09716038, cg14773523, cg14941179, and cg22299097 had a significant negative correlation with RIOX2 expression in LUAD, whereas in LUSC, methylation at cg09716038, cg14773523, cg14941179, cg22299097, cg05451573, cg10779801, and cg23629183 is negatively correlated with RIOX2 expression. According to the analysis based on the databases, RIOX2 gene could not be considered as the independent prognostic biomarker in lung adenocarcinoma or squamous cell lung cancer. However, the molecular mechanism of RIOX2 gene in the development of lung cancer may be helpful in improving lung cancer therapy.

## Introduction

Cancer is a major public health problem worldwide and an important cause of death in many countries. Lung cancer is the important cause of death in patients with malignant tumors [1]. It is generally accepted that smoking is a major risk factor for lung cancer, but in recent years, the rising incidence of non-smoking patients suggests that other factors in the development of lung cancer have played an important role, such as living environment and occupational exposure [2–4]. Lung cancer can be divided into two histological types: non-small cell lung cancer and small cell lung cancer. Non-small cell lung cancer includes squamous cell carcinoma, adenocarcinoma and large cell lung cancer. Squamous cell carcinoma usually arises from the bronchial epithelium and is most likely to occur in older men or women with a history of smoking. Adenocarcinoma mainly originates from bronchial mucous glands, which can occur in the small bronchi or the central airway. Clinically, it is mostly peripheral type, and tends to occur in women with or without smoking history. Large cell carcinoma is an undifferentiated non-small cell carcinoma, which is rare and lacks the characteristics of small cell carcinoma, adenocarcinoma or squamous cell carcinoma in cytology, histological structure and immunophenotype. About 87% of lung cancer patients were diagnosed with non-small cell lung cancer [5]. The large population of NSCLC patients also bodes well for the therapeutic value generated by the disease’s research. The social burden of lung cancer has been increasing in recent decades. Due to the lack of reliable biomarkers and corresponding clinical symptoms in the early stages of the disease, most cases are already at an advanced stage when they are discovered, with lost opportunities for surgery and poor prognosis. Therefore, early detection of lung cancer patients becomes the main target of screening. The search for valuable biomarkers associated with the disease and the aggressive development of liquid biopsies is of great significance for the early detection of patients.

Due to the limited efficacy, drug resistance and serious side effects of traditional chemotherapeutic drugs, molecular targeted drugs and immunotherapy have attracted much attention. In the past decades, the research and treatment of non-small cell lung cancer have made great progress. The rapid development of molecular targeted drug therapy and the continuous exploration of immunotherapy have great significance for prolonging the survival time of patients, especially for the treatment of advanced patients. But individualized treatment helpful for survival is limited at present. Therefore, further revealing the pathogenesis of the disease and finding new therapeutic targets are important to improve the survival rate and prognosis of patients.

Epigenetics is a branch of genetics that investigates genetic changes in gene expression without changes in the nucleotide sequence. Epigenetic changes mainly including DNA methylation, histone modification, chemical modification and chromatin remodeling, play an important role in malignant transformation of cells. DNA methylation can occur at the early stage of cancer, so it can be considered as a kind of biomarker for early cancer diagnosis, and it is more reliable than gene expression alone as a kind of biomarker for early cancer diagnosis [6]. Compared to traditional biopsy, free DNA testing is a non-invasive test that can overcome the tumor heterogeneity involved in traditional biopsy and is suitable for patients who are not medically suitable for biopsy or require multiple tissue sampling evaluations. Detection of DNA methylation in peripheral circulation or body fluid samples can make the evaluation of tumor patients more convenient and real-time. Therefore, ctDNA in plasma presents to be a promising tumor marker and can be considered to help diagnose lung cancer and guide the treatment of lung cancer by detecting DNA methylation in body fluid samples.

Ribosomal oxygenase 2 (RIOX2) is involved in gene transcription in eukaryotic cells [7]. It promotes cell proliferation, cycle transition, and anti-apoptosis carcinogenic activities [8]. Its expression is mostly up-regulated in human colon, esophagus, lung, lymphocyte, kidney, nervous system, liver, breast, pancreas, and gastric cancers [9–19]. Under normal circumstances, RIOX2 is not expressed in lung tissues but is over-expressed in non-small cell lung cancer (NSCLC) tissues [20].

Abnormal expression of RIOX2 gene was found to be associated with decreased levels of H3K9me3, H3K27me3, and H4K20me3, suggesting that the products of RIOX2 gene expression have the ability of histone demethylation. All of these indicated that RIOX2 had a demethylation enzyme-like effect on H3K9me3, H3K27me3 and H4K20me3. The effect of RIOX2 on histone demethylation has been confirmed in breast cancer cells and lung cancer cell lines [20]. This gene is a kind of key gene involved in inflammation, tumorigenesis and metastasis of tumor cells. Exploring the clinical application of gene methylation related to lung cancer can guide cancer patients to achieve better personalized treatment. A better understanding of RIOX2’s epigenetic roles, such as DNA methylation and histone modification, is beneficial to design new targeted therapies for tumors and potentially address the problem of resistance to current therapies in some patients.

Highly-expressed RIOX2 is associated with a lower overall survival rate in lung cancer patients, without lymph node metastasis or only with proximal lymph node metastasis but it does not predict that of patients with distant lymph node metastasis [21]. Patients with metastasis have been reported to have a better prognosis due to RIOX2’s over-expression [22]. However, conflicting results in the literature on the role of RIOX2 in lung cancer and its effects on prognosis have been observed.

In this article, RIOX2 expression and methylation level in lung cancer were analyzed based on the databases including the cancer genome atlas (TCGA)(https://cancergenome.nih.gov/; https://portal.gdc.cancer.gov/) and the gene expression omnibus (GEO) (https://www.ncbi.nlm.nih.gov/geo/).

## Materials and methods

### Data download

We downloaded the transcriptome data, clinical information and methylation data of squamous cell lung carcinoma and lung adenocarcinoma from the TCGA database (https://portal.gdc.cancer.gov/). We downloaded the datasets of lung adenocarcinoma and squamous cell lung carcinoma from the GEO database (https://www.ncbi.nlm.nih.gov/geo/), which include carcinoma tissues and adjacent tissues. The survival data, clinical information, and progression free survival data of lung adenocarcinoma and squamous cell lung carcinoma were downloaded from the UCSC Xena(https://xena.ucsc.edu/).

### Analysis tools

In this study, R4.0.3 software (https://www.r-project.org/) was used for data analysis and processing, involving R packages [23–32] including limma, survival, survminer, timeROC, plyr, ggpubr, reshape2, ggplot2, meta, and sva packages.

### Statistical methods

In this study, the wilcox test was used when comparing two groups and Kruskal-Wallis test was used when comparing multiple groups (3 or more groups). When multiple datasets were performed and analyzed, the analysis was performed using batch correction in consideration of laboratory factors. In this study, the Kaplan-Meier method was used to estimate the survival of patients. We divided the patients into two different groups according to the median expression level. In other words, patients with expression levels that were higher than the median were placed into the high expression group, while those with expression levels that were lower than the median were placed into a low expression group. When comparing the survival curves of two or more groups, a common test was the log-rank test. We compared them by the Breslow test. In meta-analysis, if I^2 was less than 50% and *p* was greater than 0.05, fixed-effect model was selected. If I^2 was greater than 50% and *p* was less than 0.05, the random effects model was selected. When *p* value was less than 0.05, the results could be considered statistically significant.

## Results and discussion

### Data download

#### Transcriptome data

From the TCGA database(https://portal.gdc.cancer.gov/), we obtained 551 files (normal count: 54, tumor count: 497) related to LUAD, involving 479 cases and 551 files (normal count: 49, tumor count: 502) related to LUSC, involving 501 cases.

#### Methylation data

From the TCGA database(https://portal.gdc.cancer.gov/), we obtained 466 files (29 normal and 437 tumor samples) related to LUAD, involving 425 cases and 412 files (42 normal and 370 tumor samples) related to LUSC, involving 372 cases.

### lung adenocarcinoma

#### Differential analysis on gene expression

**Fig 1:**
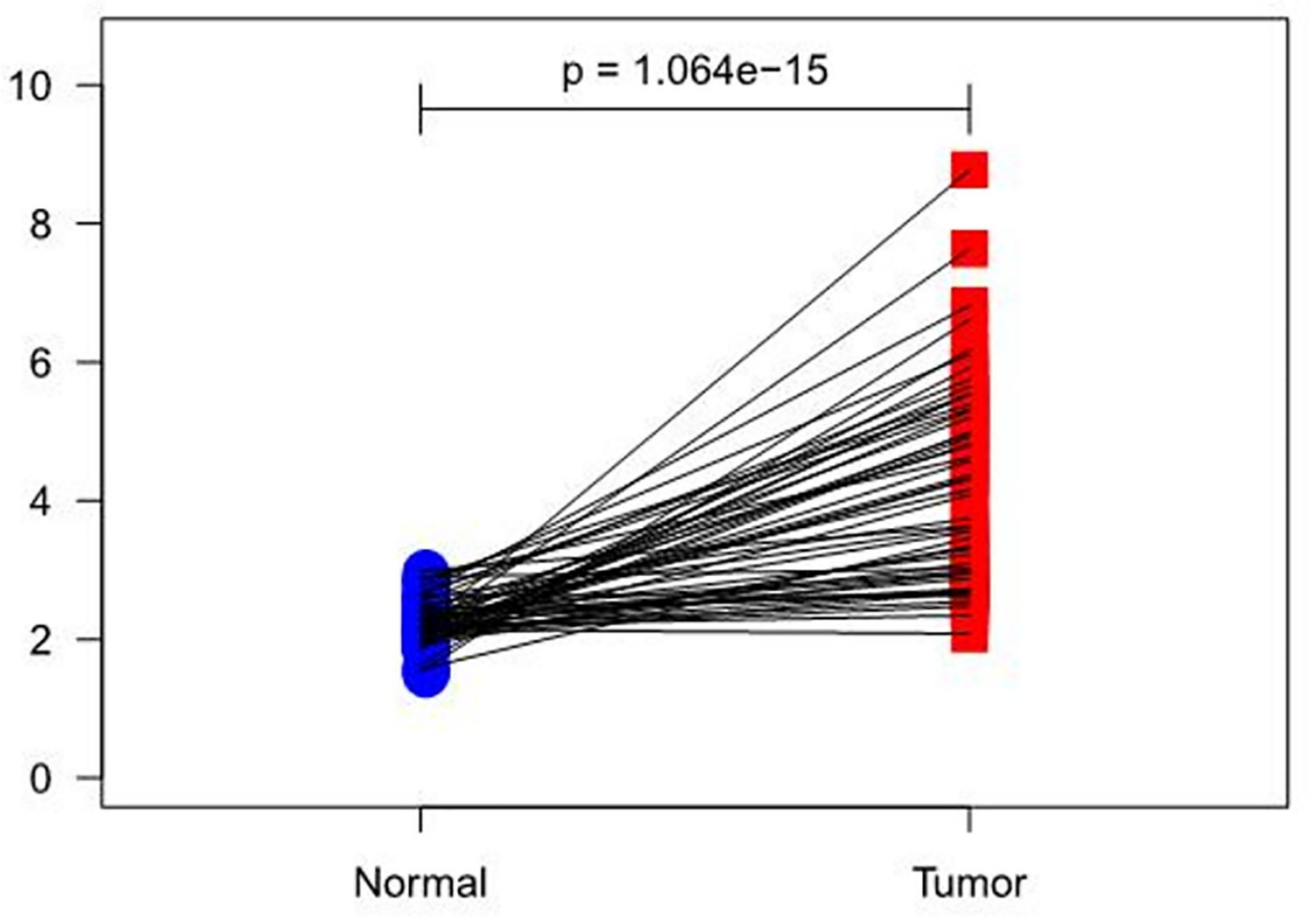
A paired scatter plot of RIOX2 gene differential expression in lung adenocarcinoma between cancerous tissues and adjacent normal tissues. The abscissa represents the sample group and the ordinate represents the RIOX2 gene expression level. The red represents the cancer tissue sample group and the blue represents the normal sample group.

Results showed that RIOX2 expression had an overall increasing trend in both groups (*p* = 1.064e-15) but it was higher in carcinoma tissues (Fig 1). Furthermore, LUAD and para-carcinoma tissue datasets were selected from the GEO database(https://www.ncbi.nlm.nih.gov/geo/). GSE85841 [33] (8 normal and 8 tumor samples) and GSE130779 [34] (8 normal and 8 tumor samples) were obtained. Results showed that logFC, logCPM, *p* value, and FDR of RIOX2 between the groups were 0.802107483, 3.681482308, 0.004666927, and 0.011185307, respectively. Taken together, we confirmed that RIOX2 expression was significantly different between LUAD and para-carcinoma tissues.

#### Differential analysis on gene methylation

From the TCGA database(https://portal.gdc.cancer.gov/), results showed that the normalMean, TumorMean, logFC, *p* value, and FDR were 0.350766084, 0.317423173, -0.144101804, 7.47E-12, and 5.42E-11, respectively.

#### Correlation analysis between expression and methylation level related to RIOX2 gene

**Fig 2:**
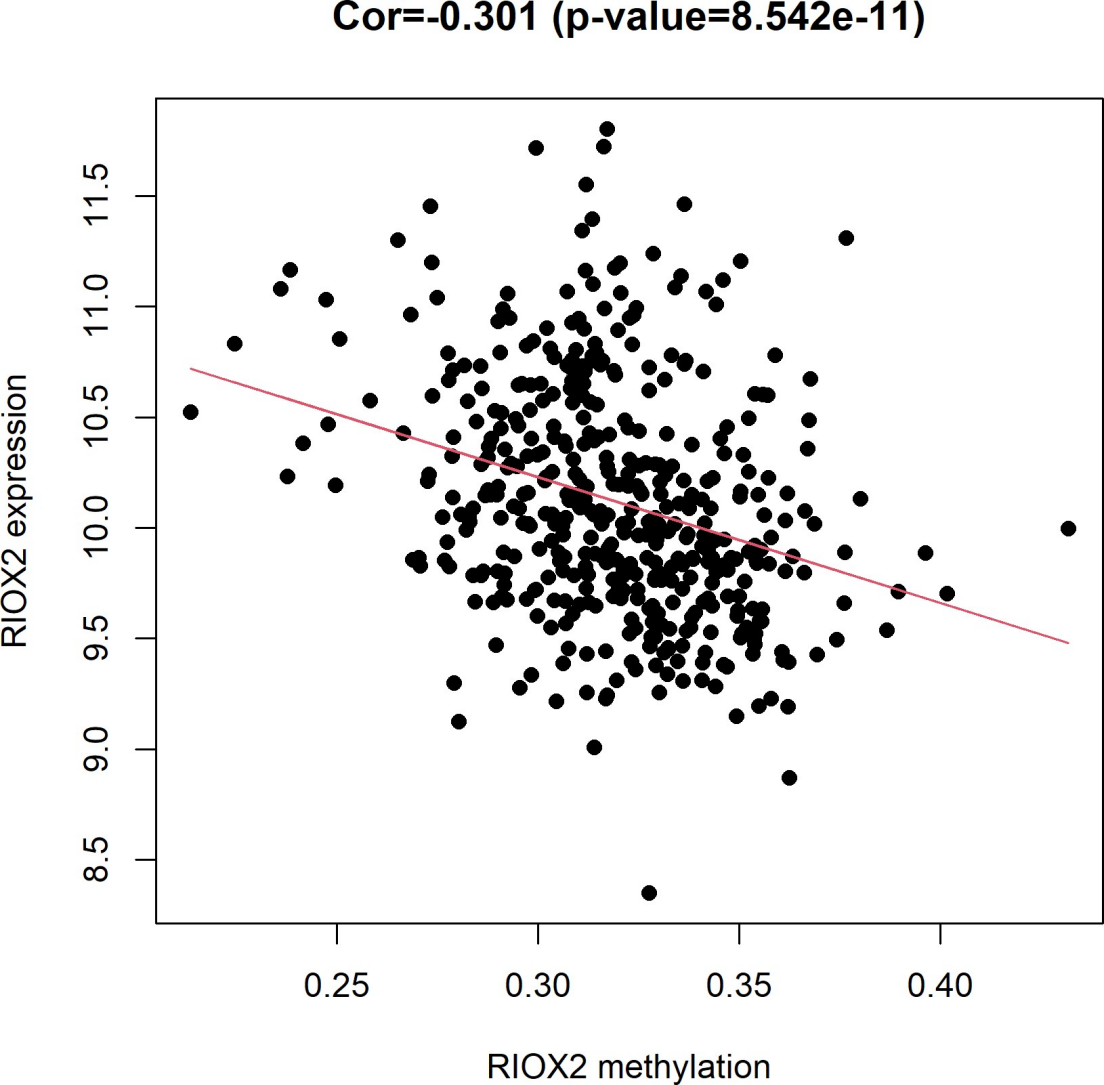
The relationship between RIOX2 gene expression and methylation levels in lung adenocarcinoma. The horizontal coordinate is the RIOX2 methylation level, and the vertical coordinate is the RIOX2 gene expression level.

From the TCGA database(https://portal.gdc.cancer.gov/), it was found that RIOX2 gene expression level had a significant (Cor = -0.301, *p* = 8.542e-11) negative correlation with the methylation level in LUAD (Fig 2).

#### Correlation analysis between RIOX2 gene expression and the methylation level of the gene sites related to RIOX2 gene

**Fig 3:**
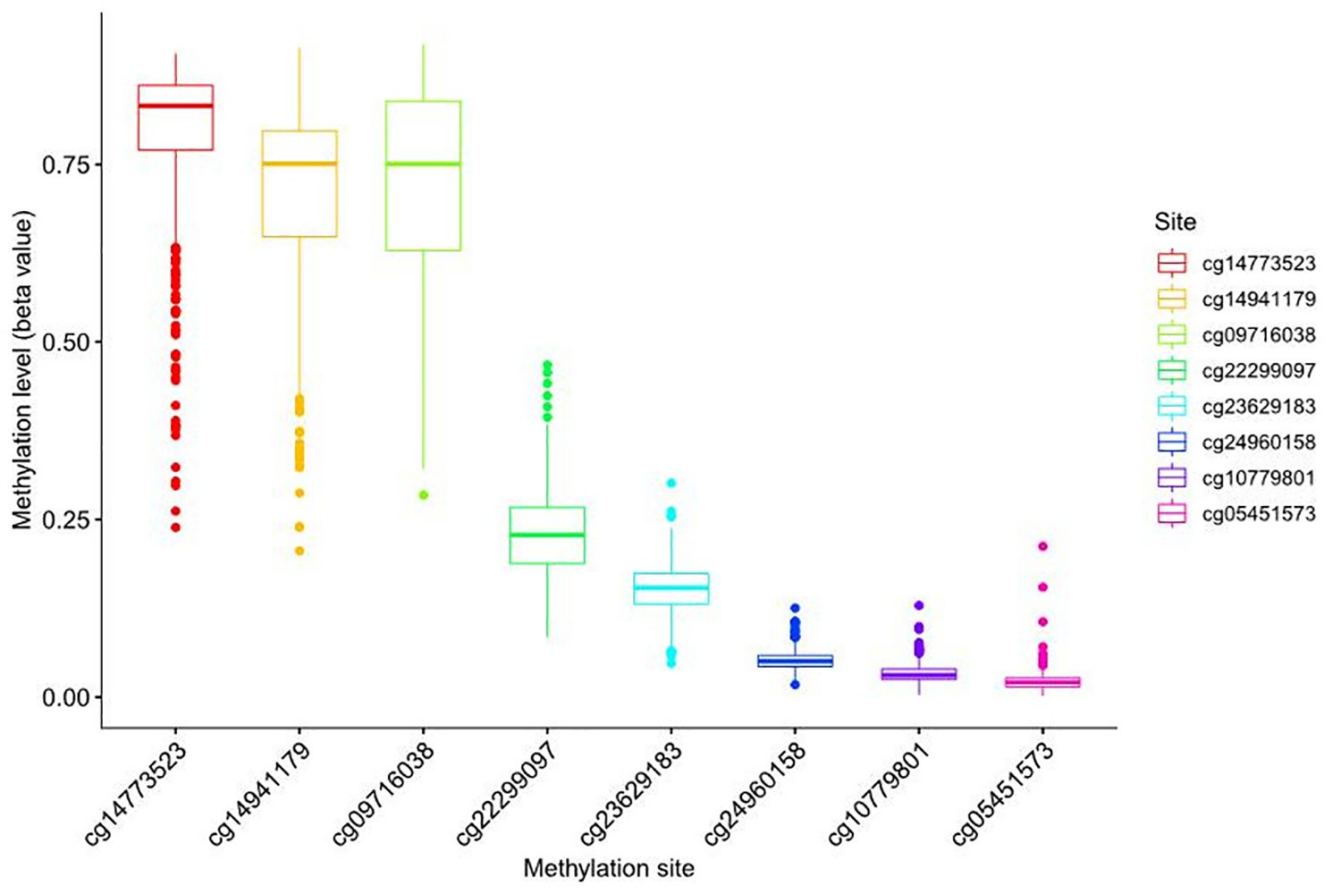
Box plot of methylation levels at RIOX2 gene-related sites in lung adenocarcinoma. The abscissa represents RIOX2 related sites, and the ordinate represents site methylation levels.

**Fig 4:**
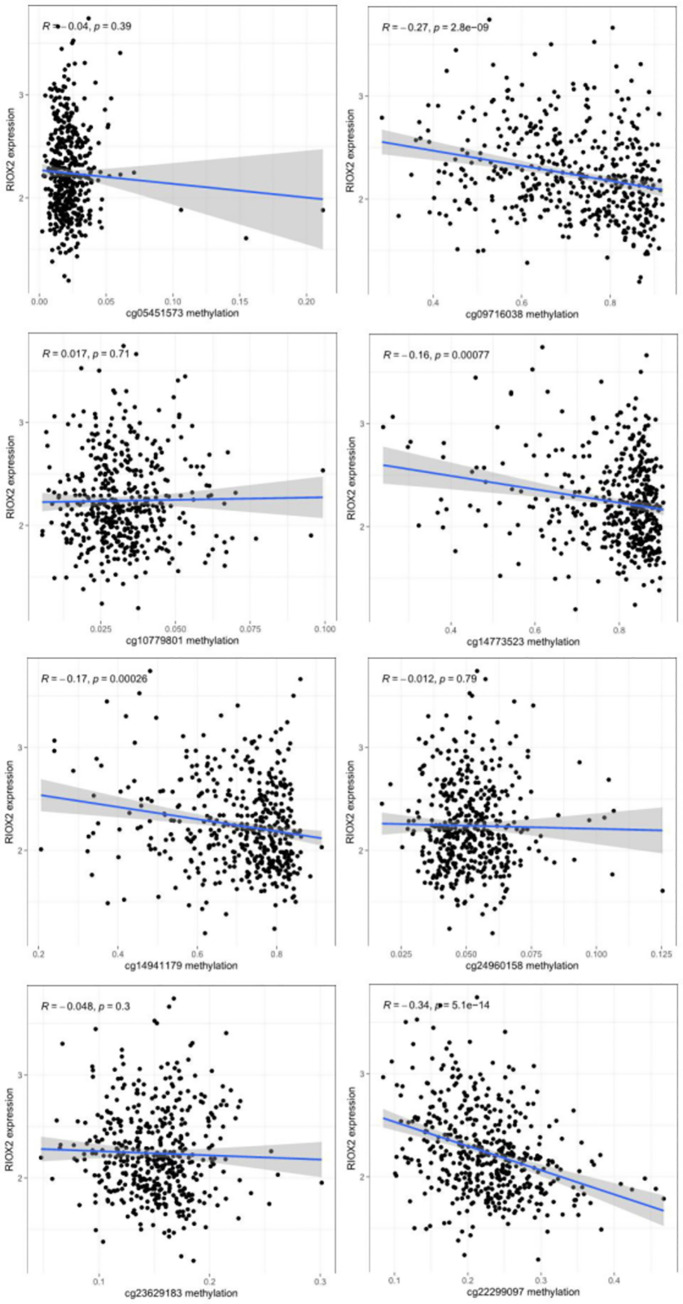
Results of correlation analysis between RIOX2 gene related site methylation and expression of RIOX2 gene in lung adenocarcinoma. The horizontal coordinate is the methylation level of RIOX2 related sites, and the vertical coordinate is the expression level of RIOX2 gene.

From the UCSC Xena (https://xena.ucsc.edu/), the box plot of site methylation in LUAD showed that the RIOX2 methylation sites included cg09716038, cg14773523, cg14941179, cg22299097, cg05451573, cg10779801, cg23629183, and cg24960158, among which the methylation level was higher at cg14773523, cg14941179, and cg09716038 (Fig 3). Furthermore, the correlation analysis between site methylation and RIOX2 expression in LUAD showed that the methylation at cg09716038, cg14773523, cg14941179, and cg22299097 had a significant negative correlation with RIOX2 expression (Fig 4).

#### RIOX2 gene expression among groups divided by different clinical traits

The gene expression, gene methylation level and patients’ clinical information of lung adenocarcinoma were obtained from the UCSC Xena(https://xena.ucsc.edu/) and grouped according to each clinical trait (age, sex, TNM stage and tumor stage). There was no significant difference in RIOX2 gene expression in groups divided by above clinical traits, but the methylation level of RIOX2 gene was different between groups of different genders. And the methylation level of RIOX2 gene was higher in female patients.

#### Survival analysis related to RIOX2 gene in lung adenocarcinoma

The survival information of lung adenocarcinoma patients was downloaded from the UCSC Xena (https://xena.ucsc.edu/) and there was no significant difference between the two groups with high and low expression of RIOX2 gene (*p* = 0.579). The analysis results about GSE68465 [35] from the GEO database (https://www.ncbi.nlm.nih.gov/geo/) revealed that the level of RIOX2 gene expression in lung adenocarcinoma was not associated with survival prognosis(*p* = 0.316).

#### Survival analysis related to RIOX2 gene methylation in lung adenocarcinoma

**Fig 5:**
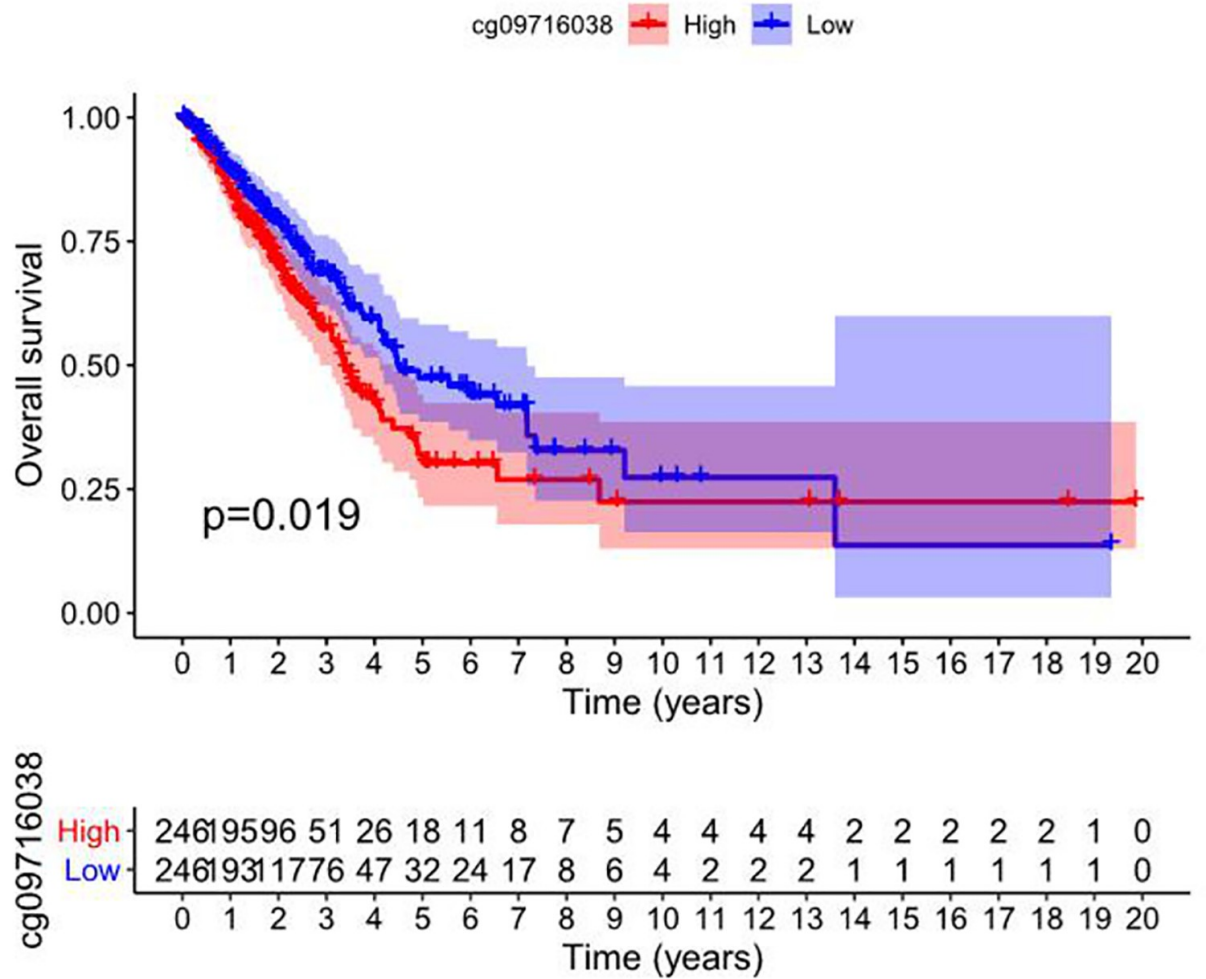
Survival analysis of high and low methylation level of cg09716038 related to RIOX2 gene in lung adenocarcinoma. The abscissa represents the survival time, and the ordinate represents the overall survival. Red represents the high methylation level of the gene site and blue represents the low methylation level of the gene site.

RIOX2 gene methylation data and patients’ survival information were downloaded from the UCSC Xena (https://xena.ucsc.edu/), and the analysis showed that the high and low methylation level of RIOX2 gene sites cg09716038 in lung adenocarcinoma was correlated with patient survival (*p* = 0.019). And the group with low methylation level at this site had a better prognosis (Fig 5).

#### PFS analysis related to RIOX2 gene methylation in lung adenocarcinoma

The methylation data of gene sites in lung adenocarcinoma and PFS information of patients was obtained from the UCSC Xena (https://xena.ucsc.edu/), and there was no significant correlation between the methylation of RIOX2 gene sites and PFS of patients with lung adenocarcinoma.

#### Meta analysis related to RIOX2 gene in lung adenocarcinoma

**Fig 6:**
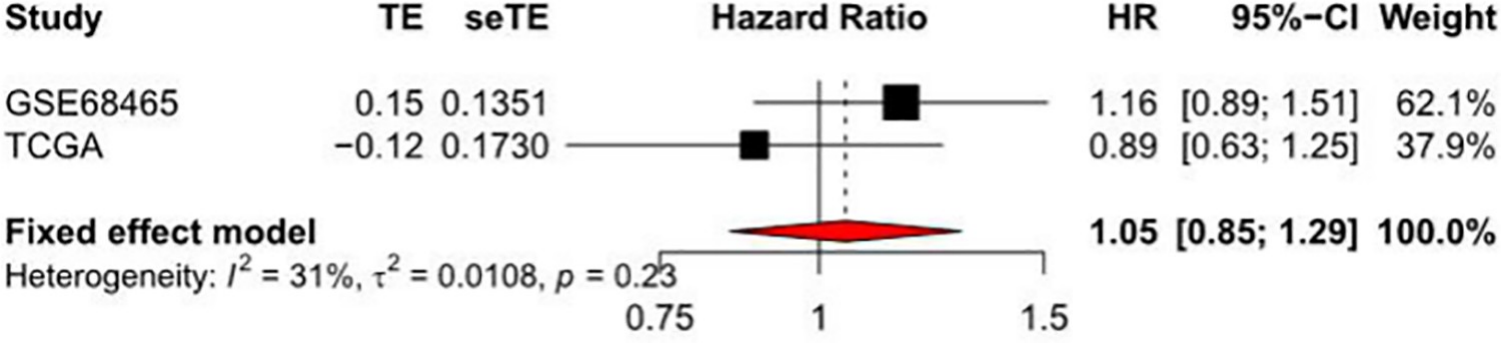
Forest plot of the hazard ratio by COX analysis related to RIOX2 gene in lung adenocarcinoma. The abscissa represents the hazard ratio and the ordinate represents the sample sources.

Meta-analysis was conducted using TCGA data obtained from the UCSC Xene (https://xena.ucsc.edu/) and datasets obtained from the GEO (https://www.ncbi.nlm.nih.gov/geo/). The HR was 1.05 (0.85,1.29), indicating that this gene has little effect on the situation of patients with lung adenocarcinoma. So this gene may not be used as a single factor to evaluate the conditions of patients (Fig 6).

### Squamous cell lung cancer

#### Differential analysis on gene expression

**Fig 7:**
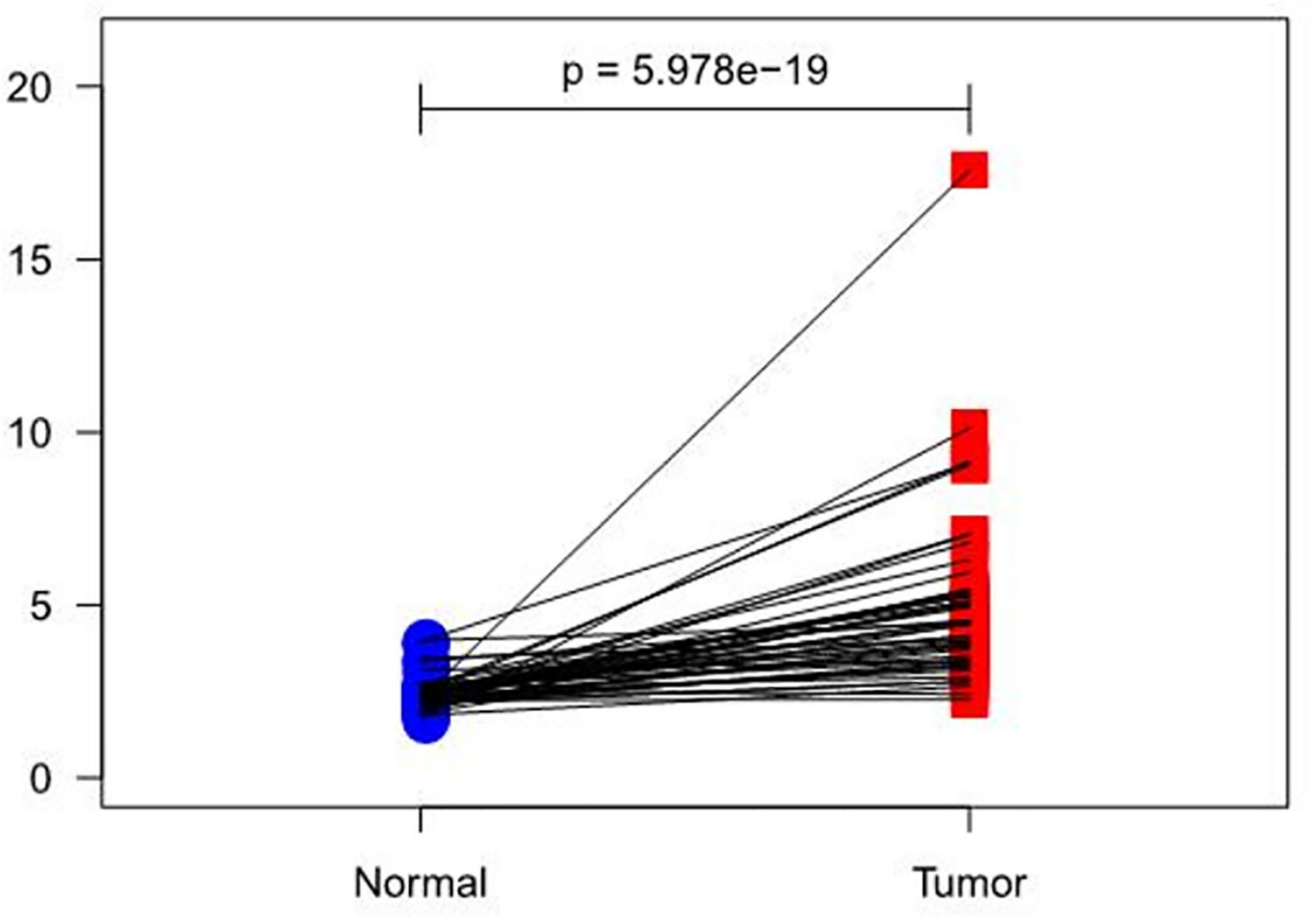
A paired scatter plot of RIOX2 gene differential expression between cancerous tissue and adjacent normal tissue in lung squamous cell carcinoma. The abscissa represents the sample group and the ordinate represents the RIOX2 gene expression level. The red represents the cancer tissue sample group and the blue represents the normal sample group.

According to the data from the TCGA database (https://portal.gdc.cancer.gov/), results showed that RIOX2 expression had an overall increasing trend in both groups (*p* = 5.978e-19) (Fig 7). However, it was higher in carcinoma tissues. In addition, LUSC and para-carcinoma tissue datasets were selected from the GEO database (https://www.ncbi.nlm.nih.gov/geo/), obtaining GSE67061 [36] (GPL6480, 69 tumor samples) and GSE101420 [37] (GPL6480, 60 normal samples) data. Results showed that logFC, logCPM, *p* value, and FDR of RIOX2 between the groups were 0.66876328, 4.874048422, 1.72E-10, and 2.37E-10 respectively.

#### Differential analysis on methylation

Based on the TCGA database (https://portal.gdc.cancer.gov/), results showed that the normalMean, TumorMean, logFC, *p* value, and FDR in LUSC were 0.341910847, 0.318043133, -0.104397754, 1.15E-13 and 4.60E-13, respectively.

#### Correlation analysis between expression and methylation level related to RIOX2 gene

**Fig 8:**
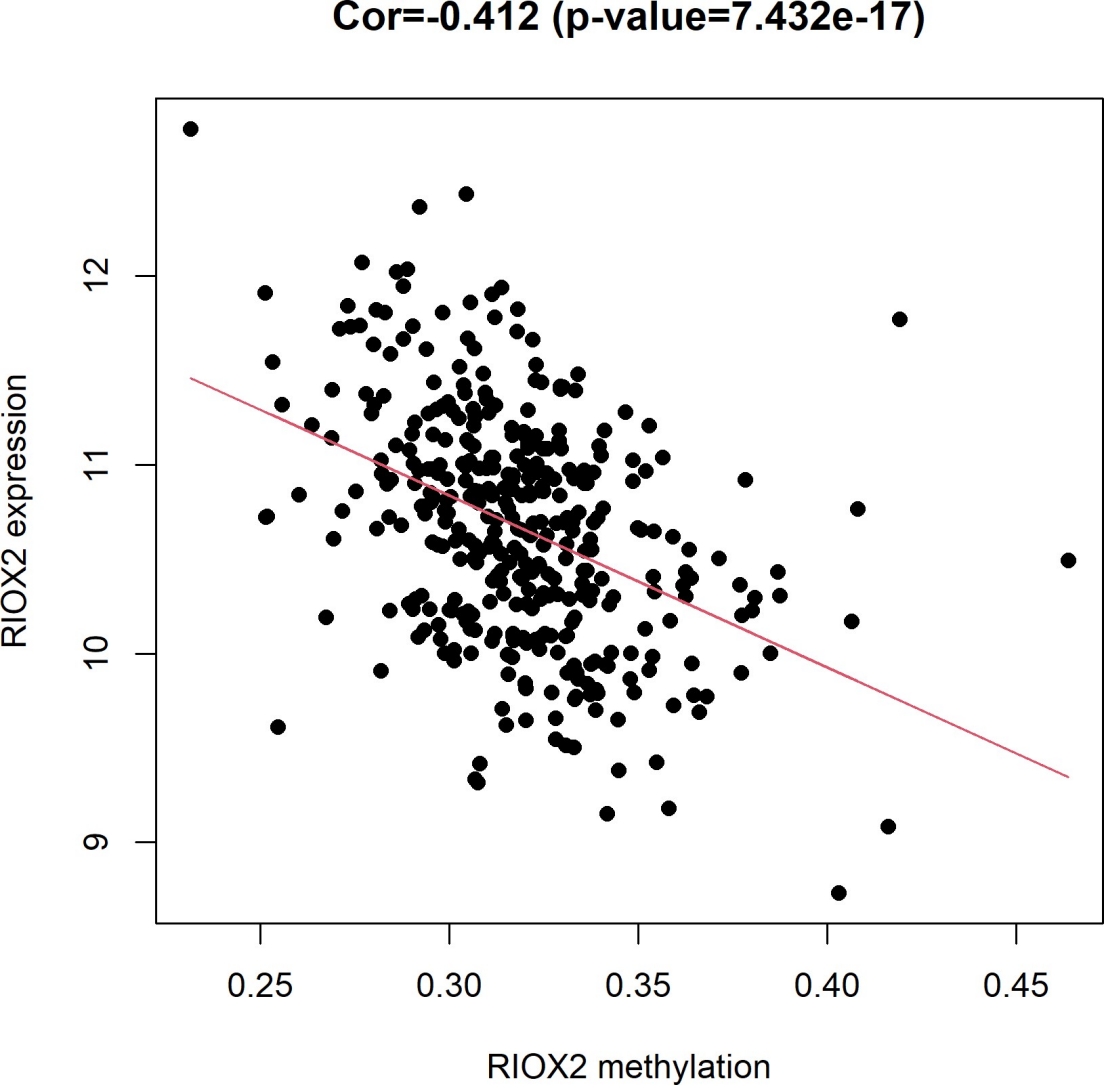
The relationship between RIOX2 gene expression and methylation levels in lung squamous cell carcinoma. The horizontal coordinate is the RIOX2 methylation level, and the vertical coordinate is the RIOX2 gene expression level.

According to the data obtained from the TCGA(https://portal.gdc.cancer.gov/), it was found that gene expression level had a significant (Cor = -0.412, *p* = 7.432e-17) negative correlation with the methylation level (Fig 8).

#### Correlation analysis between RIOX2 gene expression and the methylation level of the gene sites related to RIOX2 gene

**Fig 9:**
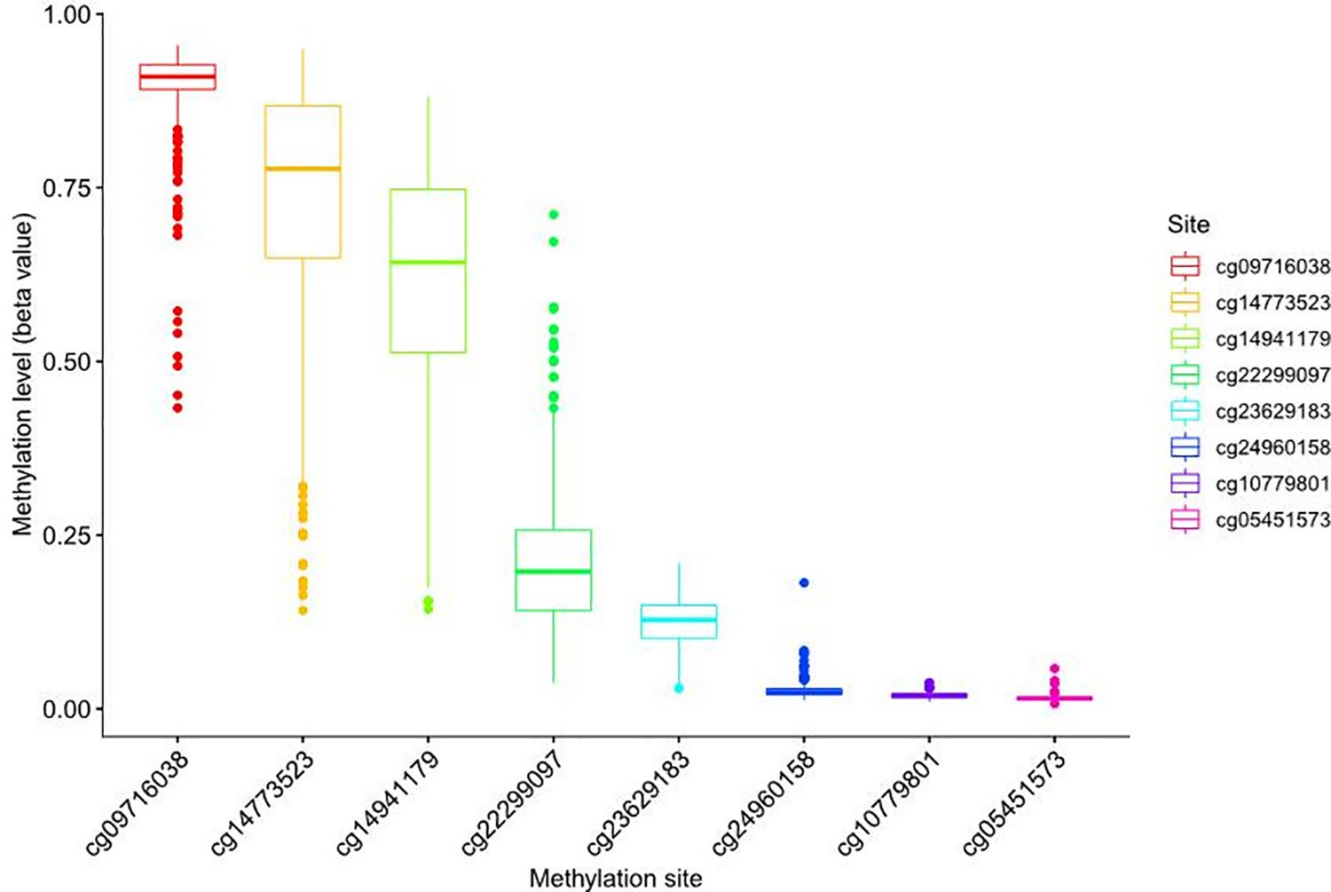
Box plot of methylation levels at RIOX2 gene-related sites in lung squamous cell carcinoma. The abscissa represents RIOX2 related sites, and the ordinate represents site methylation levels.

**Fig 10:**
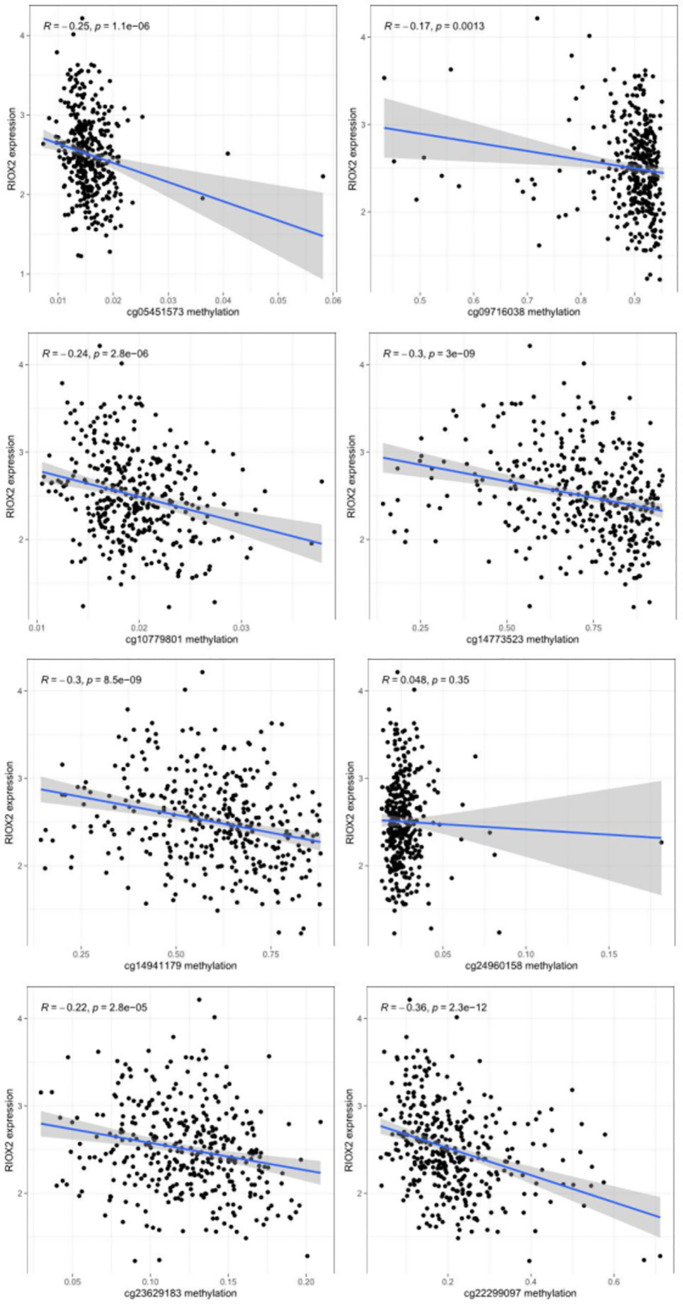
Results of correlation analysis between RIOX2 gene related site methylation and expression of RIOX2 gene in lung squamous cell carcinoma. The horizontal coordinate is the methylation level of RIOX2 related sites, and the vertical coordinate is the expression level of RIOX2 gene.

Data on RIOX2 methylation were extracted from those obtained from the UCSC Xena (https://xena.ucsc.edu/), the box plot of site methylation in LUSC showed that the RIOX2 methylation sites included cg09716038, cg14773523, cg14941179, cg22299097, cg05451573, cg10779801, cg23629183, and cg24960158, among which the methylation level was higher at cg09716038, cg14773523, and cg14941179 (Fig 9). Among data obtained from the UCSC Xena(https://xena.ucsc.edu/), results showed that methylation at cg09716038, cg14773523, cg14941179, cg22299097, cg05451573, cg10779801, and cg23629183 had a negative correlation with RIOX2 expression (Fig 10).

#### RIOX2 gene expression among groups divided by different clinical traits

Gene expression, gene methylation level and clinical information of patients with squamous cell lung carcinoma were obtained from the UCSC Xena (https://xena.ucsc.edu/). The analysis result showed that RIOX2 gene expression and methylation level were different in groups with the different conditions of lymph node metastasis among patients with squamous cell lung carcinoma. There were significant differences in gene expression and methylation level between N1 and N2 (*p* = 0.015, *p* = 0.014, respectively), and the gene expression level in N2 was higher, while its methylation level was lower. The methylation level of this gene was significantly different between different genders (*p* = 0.047), and the gene methylation level was higher in female patients. There were significant differences in gene methylation levels among patients of different ages (*p* = 0.023), and the gene methylation levels were higher in patients who were less than 50 years old.

#### Survival analysis related to RIOX2 gene in squamous cell lung cancer

The survival information and gene expression information of patients with squamous cell lung cancer were downloaded from the UCSC Xena (https://xena.ucsc.edu/), and the analysis result showed that there was no significant difference between groups with the high and low expression of RIOX2 gene(*p* = 0.684). The datasets GSE73403 [38] and GSE157010 [39] from the GEO database (https://www.ncbi.nlm.nih.gov/geo/) showed the level of gene expression in squamous cell lung cancer has nothing to do with the prognosis of patients (*p* = 0.723, *p* = 0.758).

#### Survival analysis related to RIOX2 gene methylation in squamous cell lung cancer

The methylation data and survival information of patients with squamous cell lung cancer were obtained from the UCSC Xena (https://xena.ucsc.edu/), and the results showed that there was no significant correlation between RIOX2 gene-related methylation sites and the prognosis of patients with squamous cell lung cancer.

#### PFS analysis related to RIOX2 gene methylation in squamous cell lung cancer

**Fig 11:**
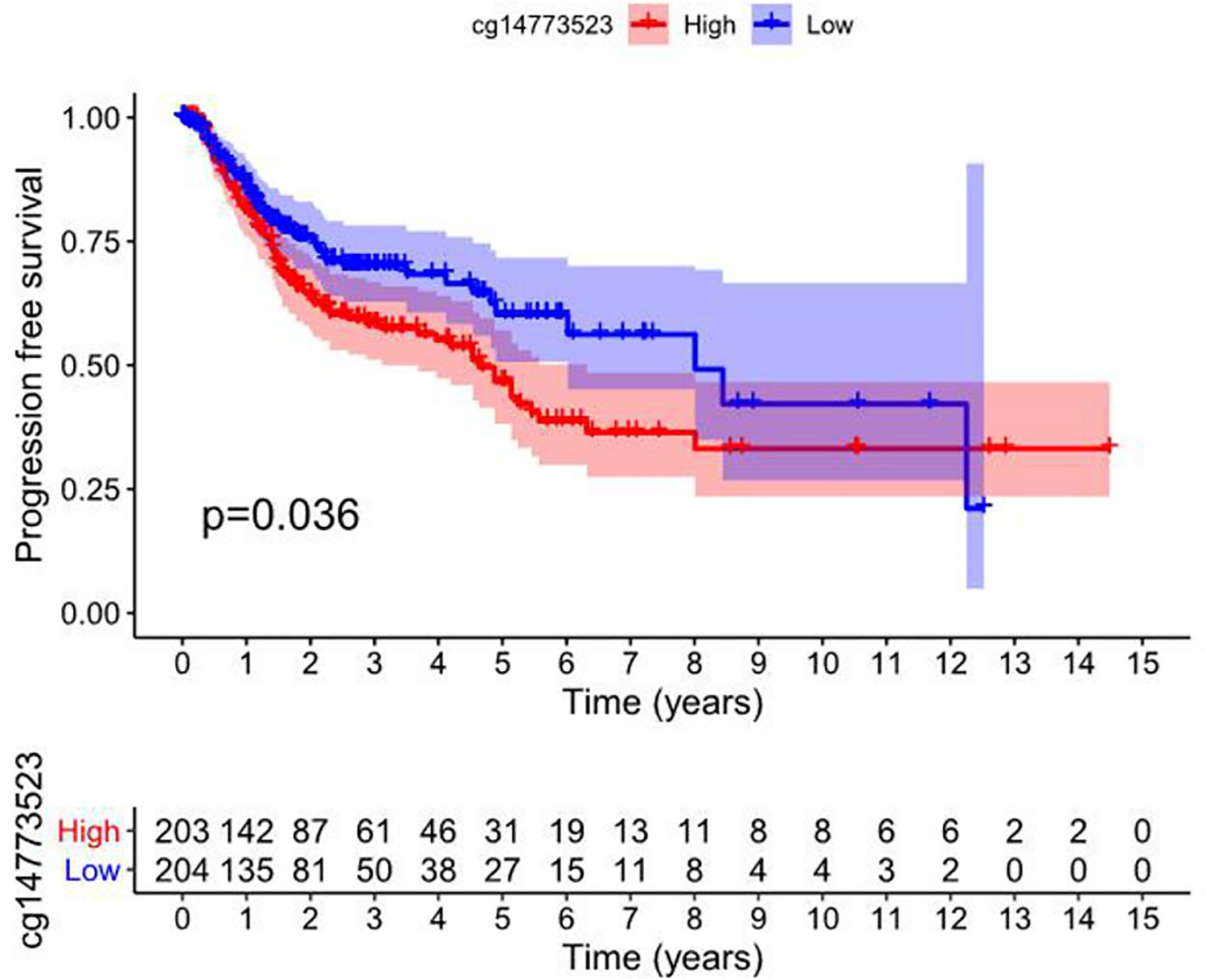
Survival analysis of high and low methylation level of cg14773523 related to RIOX2 gene in squamous cell lung cancer. The abscissa represents the survival time and the ordinate represents the progression-free survival. Red represents high methylation level of the gene site and blue represents low methylation level of the gene site.

The methylation data of squamous cell lung cancer and PFI information of patients were obtained from the UCSC Xena (https://xena.ucsc.edu/). The results showed that the high or low methylation level of RIOX2 gene site cg14773523 was correlated with progression-free survival of patients with squamous cell lung cancer and there was significant difference between groups (*p* = 0.036) (Fig 11).

#### Meta analysis related to RIOX2 gene in squamous cell lung cancer

**Fig 12:**
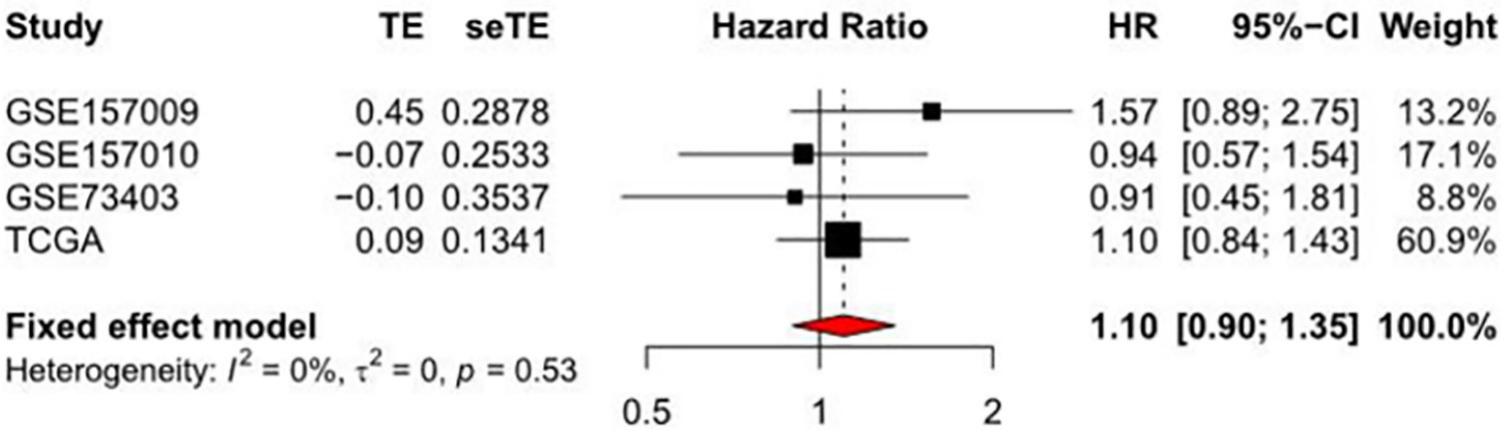
Forest plot of the hazard ratio by COX analysis related to RIOX2 gene in squamous cell lung cancer. The abscissa represents the hazard ratio and the ordinate represents the sample sources.

Meta-analysis was conducted using TCGA data obtained from the UCSC Xena (https://xena.ucsc.edu/) and datasets [38, 39] including GSE157009, GSE157010, GSE73403, which were obtained from the GEO (https://www.ncbi.nlm.nih.gov/geo/). The HR was 1.10 (0.90, 1.35), indicating that this gene has little effect on the conditions of patients with squamous cell lung cancer. So this gene may not be used as a single factor to evaluate the conditions of patients (Fig 12).

## Conclusions

In the genome of healthy people, CpG sites located in CpG islands are usually in a non-methylated state, whereas those outside are commonly found in a methylated state. Such methylation is stably retained and inherited during cell division [21]. Tumorigenesis may be related to hypomethylation by decreased methylation level of oncogenes or hypermethylation of CpG sequences in CpG islands in some cancer suppressor genes. Changes in DNA methylation have been reported in atypical adenomatous hyperplasia [22]. Such alterations are considered as an early event in the occurrence and development of tumors [40, 41], thus functioning as biomarkers for early diagnosis of cancer. Furthermore, they are more reliable in tumor prediction than simple changes in gene expression [42].

Liquid biopsies are performed to obtain free DNA from blood, urine, and other body fluid samples in real time, and assess DNA expression or methylation using the captured molecules. They are useful to detect early tumors and evaluate their condition, prognosis, and treatment [43]. Furthermore, liquid biopsy is appropriate for patients who are not suitable for tissue biopsy due to physical conditions or those who require multiple tissue sampling. In this regard, RAPSN hypomethylation in peripheral blood and lung cancer tissues was associated with early carcinogenesis [44], whereas others reported that detection of methylation gene promoters in sputum samples improved the accuracy of early diagnosis of lung cancer and performed better that using plasma samples [45]. Lung cancer is diagnosed early and more accurately in patients with lung nodules by DNA methylation biomarkers in blood or urine samples, combined with CT examination [46]. Moreover, DNA methylation may have the potential to improve the clinical outcome of patients and its sites may serve as biomarkers for predicting the patient’s response to immunotherapy [47].

The methylation levels of promoters and enhancers are related to the effect of anti-PD-1 immunotherapy on NSCLC patients [48]. Related treatments and immunological preparations in combination with epigenetic changes are helpful for immune checkpoint inhibitor-resistant people [49], however additional studies are still needed. This study analyzed RIOX2 gene based on TCGA and GEO databases. However, as the gene has not been applied in clinical testing at present, the collection of clinical samples is difficult and limited. The results analyzed in this study need to be verified by large-sample clinical and laboratory studies in the future.

The expression and methylation characteristics of RIOX2 in lung cancer indicate its unique role in tumor progression, which affects the prognosis of patients. Research on RIOX2 may then result in improved individualized treatment strategies in lung cancer patients.
